# Prevalence of Metabolic Syndrome and Framingham Risk Score in Apparently Healthy Vegetarian and Omnivorous Men

**DOI:** 10.5935/abc.20180073

**Published:** 2018-05

**Authors:** Julio Cesar Acosta Navarro, Luiza Antoniazzi, Adriana Midori Oki, Maria Carlos Bonfim, Valeria Hong, Luiz Aparecido Bortolotto, Pedro Acosta-Cardenas, Valeria Sandrim, Marcio Hiroshi Miname, Raul Dias dos Santos Filho

**Affiliations:** 1 Instituto do Coração (InCor) - Faculdade de Medicina da Universidade de São Paulo, São Paulo, SP - Brazil; 2 Hospital Regional de Ferraz de Vasconcelos - Osiris Florindo,São Paulo, SP - Brazil; 3 Departamento de Farmacologia do Instituto de Biociências de Botucatu - Universidade Estadual Paulista (UNESP), São Paulo, SP - Brazil

**Keywords:** Metabolic Syndrome, Coronary Artery Disease, Vegetarians, Men, Risk Factors, Diet, Vegetarian

## Abstract

**Background:**

Recent studies have shown a lower prevalence of metabolic syndrome (MSyn) in vegetarians (VEG) despite the inconclusive evidence from others.

**Objective:**

To verify the association between diet and other lifestyle characteristics and the prevalence of MSyn, cardiovascular risk factors (CRF), and Framingham Risk Score (FRS) in apparently healthy VEG and omnivorous (OMN) men.

**Methods:**

In this cross-sectional study, 88 apparently healthy men ≥ 35 years, 44 VEG and 44 OMN, were assessed for anthropometric data, blood pressure, blood lipids, glucose, C-reactive protein (CRP) and FRS. To test the association between lifestyle and MSyn, Student t test, chi-square test, and multiple logistic regression model were used. A significance level of 5% was considered in all statistical analyses.

**Results:**

Several CRF were significantly lower in VEG than in OMN: body mass index, systolic blood pressure, diastolic blood pressure, fasting serum total cholesterol, LDL-cholesterol, apolipoprotein b, glucose, and glycated hemoglobin (all p < 0.05). The FRS mean was lower in VEG than in OMN (2.98 ± 3.7 vs 4.82 ± 4.8, p = 0.029). The percentage of individuals with MSyn was higher among OMN than among VEG (52.3 vs.15.9%) (p < 0.001). The OMN diet was associated with MSyn (OR: 6.28 95%CI 2.11-18.71) and alterations in most MSyn components in the multiple regression model independently of caloric intake, age and physical activity.

**Conclusion:**

The VEG diet was associated with lower CRF, FRS and percentage of individuals with MSyn.

## Introduction

The number of individuals consuming a vegetarian (VEG) or plant-based diet is increasing, and there is evidence that this habit is associated with a lower prevalence of risk factors for cardiovascular diseases (CVD).^1^^-^^5^ A few studies in the literature have evaluated the association between the VEG diet and the lower risk of coronary heart disease (CHD),^5^^,^^6^ according to the Framingham Risk Score (FRS), an algorithm for assessing risk for CHD in the short term (< 10 years).^7^ Furthermore, recent studies have shown a lower prevalence of metabolic syndrome (MSyn) in VEG^8^^,^^9^ despite the inconclusive evidence from others.^10^^-^^14^ The only study found in the scientific literature that evaluated the relationship in a Latin American population has demonstrated no association.^5^ The importance of MSyn rests in the fact that approximately one in four adults in the United States has MSyn, which is considered a risk factor for atherosclerotic cardiovascular disease.^15^ In addition, 20-25% of adults worldwide have MSyn, which doubles the risk for having a heart attack and triples the risk for stroke,^16^^-^^18^ in addition to increasing the risk of death in the general population.^19^ The aim of this observational study was to investigate the association between the type of diet and the prevalence of MSyn assessed in apparently healthy VEG and omnivorous (OMN) men. Our hypothesis is that VEG men have better indicators of these conditions compared to OMN men.

## Methods

In the recently published cross-sectional *Carotid Atherosclerosis and Arterial Stiffness in Vegetarians and Omnivorous Subjects* (CARVOS) study,^20^ 745 adult volunteers initially were recruited in São Paulo through social activities and the Internet. The participants filled out questionnaires regarding past medical history, family history, dietary preferences, and personal data. Exclusion criteria were: 1) being female 2) history of diabetes; 3) history of dyslipidemia; 4) history of CVD or cerebrovascular diseases; 5) history of hypertension or intake of antihypertensive medication; and 6) smoking. All individuals who declared themselves to be “smokers” or “occasional smokers” at the interview or had quit smoking in the last month prior to the interview were considered smokers.

Although the exclusion criteria of the research project were related to factors that are MSyn components, they were the reference of the individual on previous diagnosis, and it was verified that several individuals presented MSyn, enabling the development of the present study, which aims to compare the percentage of individuals with MSyn in the two groups according to the type of diet.

Healthy participants ≥ 35 years were divided into two groups - VEG and OMN - according to their dietary patterns. Vegetarian men were defined as exclusively consuming a vegetarian diet void of meat, fish, and poultry for at least four years; these men could be lacto-ovo-vegetarians (consuming eggs, milk, and dairy products), lacto-vegetarians (consuming milk and dairy products) or vegans (consuming no eggs or milk or dairy products). Matched OMN men were defined as consuming any type of meat at least four or more servings per week.

From June 2013 to January 2014, after applying inclusion and exclusion criteria, 88 apparently healthy men were enrolled in the study (44 VEG and 44 OMN).

All 88 subjects were screened for health status with questionnaires regarding educational level, personal data, past medical history, smoking status and habitual alcohol consumption (yes or no).

Systolic blood pressure (SBP) and diastolic blood pressure (DBP) were measured twice in the right arm after a 10-minute rest in a supine position using a calibrated and averaged digital sphygmomanometer.

Subjects were interviewed, and the average of two 24-hour dietary recalls (one on weekdays and one on weekends) was used to estimate daily consumption of different nutrients. A database for Brazilian food composition was used to calculate the daily energy and nutrient intake.^21^

For measuring weight, we used a 150-kg platform scale (Filizola^®^) with 100-gram divisions. The patient was positioned in the center of the scale, standing barefoot, wearing as little clothing and as few accessories as possible. To measure height, a portable stadiometer was used, positioned in an appropriate place, with the barefoot volunteer with feet together, standing erect, with the back of the head, shoulders, buttocks, calves and heels touching the wall, and the head in the Frankfurt horizontal plane (imaginary line from the external auditory canal to the lower eye socket).^22^

Body mass index (BMI) was calculated by dividing body weight (kg) by the square of height (m).

To measure waist circumference (WC), the individual remained upright with arms relaxed along the body, with the region for measurement unclothed. For WC, the measurement was made with a tape measure at the midpoint between the last rib and the iliac crest, with the abdomen relaxed, at the end of expiration.^23^

All measures were performed in triplicate, and the mean value was used for analysis.

After fasting for 10-12 hours overnight, participants had blood samples drawn from the antecubital vein. Serum lipids, including triglycerides (TG), total cholesterol (TC), and high-density lipoprotein cholesterol (HDL-c), were assayed by using enzymatic methods with an automatic multichannel chemical analyzer (Siemens Healthcare, Newark, USA) in the central laboratory of the InCor. Low-density lipoprotein cholesterol (LDL-c) was calculated using the Friedewald formula.^24^

Glycosylated hemoglobin (HbA1c) was determined using the immunoturbidimetric method certified by the NGSP-National Glycohemoglobin Standardization Program, using the Flex kit (Siemens Healthcare, Newark, USA). For apolipoprotein b (Apo b) and fasting glucose measurements, blood samples were centrifuged at 3000 rpm for 15 minutes within 60 minutes of collection and stored at −70ºC until analysis. Fasting serum glucose (FSG) was determined by the glucose oxidase method using a Dimension RXL (Siemens Healthcare, Newark, NJ, USA) through standard laboratory techniques. Quality control assessment was performed daily for all determinations.

Subjects reported activity levels using the International Physical Activity Questionnaire-Short Form (IPAQ),^25^ which measures leisure time, domestic, work-related, and transport-related physical activities. Four domains were measured: sitting, walking, moderate-intensity activity, and vigorous-intensity activity during the previous seven days.

For analysis, we considered the following categorization: physically active (≥ 20 minutes/vigorous activity sessions ≥ 3 days/week; and/or ≥ 30 minutes/moderate activity sessions or hiking ≥ 5 days/week; and/or ≥ 150 minutes/week of any added activities - vigorous or moderate or walking), and irregularly active (<150 minutes/week of any added activities - vigorous or moderate or walking).^26^

Metabolic syndrome (MSyn) was defined according to the criteria of the International Diabetes Federation (IDF), that considers that an individual with MSyn must have central obesity (defined as WC with ethnicity specific values) plus any two of the following four factors: TG ≥ 150 mg/dL (1.7 mmol/L) or specific treatment for this lipid abnormality, HDL-c < 40 mg/dL (1.03 mmol/L) in males or specific treatment for this lipid abnormality, SBP ≥ 130 mm Hg or DBP ≥ 85 mm Hg or treatment of previously diagnosed hypertension, FSG ≥ 100 mg/dL (5.6 mmol/L) or previously diagnosed type 2 diabetes.^16^^,^^17^

For South and Central Americans, the IDF recommends the use of South Asian WC values until more specific data are available. Thus, this study considered the WC value to be ≥ 90 cm.^18^ The BMI was categorized according to the values suggested by the World Health Organization.^27^

The Framingham Heart Study provides an algorithm for assessing risk for CHD in the short term (≤ 10 years). The FRS classifies the individual CHD risk based on assigned points for age, TC, HDL-c, smoking status, SBP, and the use of medication to treat high blood pressure. The results of the FRS range are from 1% to 30% of CHD risk in 10 years.^7^

### Statistical analysis

The continuous variables were tested by the Kolmogorov-Smirnov test and presented Gaussian distribution, being demonstrated in means ± standard deviation (SD). Unpaired Student's *t* test was used for testing differences for numerical variables. The chi-square test was used to compare categorical variables between groups. Level of significance was set p < 0.05.

To test the association between the type of diet (OMN or VEG) and MSyn and its components, multiple logistic regression was used. The magnitude of effect was measured by using the OR (odds ratio) and respective 95% confidence interval (95%CI). Univariate analysis and variables with p < 0.20 were included in the multiple regression, and adjusted for caloric intake, age, physical activity level, and alcohol consumption. All analyses were performed using Stata 10.0.

## Results

No difference was found in age between the VEG and OMN groups. Vegetarians had significantly lower values for BMI, WC, SBP, DBP, TC, LDL-c, Apo b, TG, TC/HDL-c ratio, FSG, and HbA1c. Most of the individuals had less than 10 points on the FRS, only three VEG and eight OMN had FRS 10 to 20 points. No statistical difference was found when this distribution was compared by categories, but the CHD risk evaluated by FRS was higher in OMN based on a comparison of the mean score between the two groups (Table 1).

**Table 1:** Anthropometric, clinical and biochemical characteristics of apparently healthy vegetarian and omnivorous men

	Vegetarian (n = 44)	Omnivorous (n = 44)	p
Age	45.5 ± 7.8	46.8 ± 9.6	0.23
BMI (kg/m^2^)	23.1 ± 2.9	27.2 ± 4.8	< 0.001
WC (cm)	84.9 ± 7.71	95.7 ± 13.8	< 0.001
SBP (mm Hg)	119.5 ± 10.4	129.2 ± 15.1	< 0.001
DBP (mm Hg)	75.2 ± 8.6	83.9 ± 10.4	< 0.001
TC (mg/dL)	180.1 ± 40.5	202.7 ± 35.3	0.003
LDL-c (mg/dL)	110 ± 33.2	128.5 ± 32.4	0.005
Apo b (mg/L)	0.88 ± 0.28	1.01 ± 0.26	0.009
TG (mg/dL)	112.2 ± 72.2	143.9 ± 64	0.016
HDL-c (mg/dL)	47.6 ± 9.3	45.5 ± 11.6	0.17
TC/HDL-c ratio	4.0 ± 1.3	4.7 ± 1.3	0.005
FSG (mg/dL)	94.8 ± 7.2	102.9 ± 13.1	< 0.001
HbA1c (%)	5.3 ± 0.3	5.5 ± 0.5	0.004
FRS	2.98 ± 3.70	4.82 ± 5.17	0.029

Data are means ± SD. Significant values for p < 0.05. Unpaired Student's t-test. BMI: body mass index; WC: waist circumference; SBP: systolic blood pressure; DBP: diastolic blood pressure; TC: total cholesterol; LDL-c: low-density lipoprotein cholesterol; Apo b: apolipoprotein b; TG: triglycerides; HDL-c: high-density lipoprotein cholesterol; FSG: fasting serum glucose; HbA1c: glycosylated hemoglobin; FRS: Framingham Risk Score.

Although there was no significant difference in caloric intake between the two groups, VEG consumed significantly more carbohydrates (63.2 vs. 51.9% of energy, p > 0.001), dietary fiber (28.2 vs 17.9 g, p < 0.001), and polyunsaturated fat (4.0 vs. 2.7% of energy, p = 0.004) than OMN did. Moreover, OMN ingested significantly greater amounts of protein (19.5 vs. 17.1% of energy, p = 0.04), total fat (29.1 vs. 24.8% of energy, p = 0.006), saturated fat (6.9 vs. 4.4% of energy, p < 0.001), and monounsaturated fat (6.8 vs. 4.5% of energy, p < 0.001) (Table 2).

**Table 2:** Pattern of energy and nutrient ingestion of apparently healthy vegetarian and omnivorous men.

Energy and nutrient ingestion	Vegetarian (n = 44)	Omnivorous (n = 44)	p
Energy (kcal)	2,177 ± 559	2,348 ± 736	0.11
Protein (% of energy)	17.1 ± 7.8	19.5 ± 4.5	0.04
Carbohydrates (% of energy)	63.2 ± 11.6	51.9 ± 9.7	< 0.001
Total Fat (% of energy)	24.8 ± 8.3	29.1 ± 7.2	0.006
Saturated fat (% of energy)	4.4 ± 3.2	6.9 ± 2.9	< 0.001
Mono-unsaturated fat (% of energy)	4.5 ± 2.4	6.8 ± 2.8	< 0.001
Polyunsaturated fat (% of energy)	4.0 ± 2.7	2.7 ± 1.6	0.004
Cholesterol (mg)	69.3 ± 224	258.1 ± 169	< 0.001
Fiber (g)	28.2 ± 15.9	17.9 ± 13.6	< 0.001

Data are means ± SD. Significant values for p < 0.05. Unpaired Student's t-test.

Most individuals had ≥ 8 years of schooling (83.2%), but a higher percentage of OMN (30.8%) had less than 8 years of schooling compared to VEG (4.6%) (p = 0.001). Considering physical activity assessed by IPAQ, a significantly greater number of VEG was classified as physically active (n = 36, 81.8%) compared to OMN (n = 25, 56.8%; p = 0.011). Regarding alcohol consumption, 43.2% of VEG (n = 19) and 59.1% (n = 26) of OMN reported drinking alcohol, but without a statistical difference (p = 0.14).

Considering the MSyn proposed by the IDF, there were more OMN (52.3%) than VEG (15.9%) with MSyn (p < 0.001). OMN had a significantly higher occurrence of abnormal values for most of the MSyn components, WC, TG, FSG, SBP, and DBP, as shown in Table 3.

**Table 3:** Distribution of individuals with metabolic syndrome and inadequacy of its components in apparently healthy vegetarian and omnivorous men

	Vegetarian (n = 44) % (n)	Omnivorous (n = 44) % (n)	p
MSyn	15.9 (7)	52.3 (23)	< 0.001
WC (≥ 90 cm)	20.5 (9)	63.6 (28)	< 0.001
SBP (≥ 130 mm Hg)	22.7 (10)	45.5 (20)	0.025
DBP (≥ 85 mm Hg)	13.6 (6)	40.9 (18)	0.004
TG (≥ 150 mg/dL)	25.0 (11)	45.5 (20)	0.045
HDL-c (< 40 mg/dL)	22.7 (10)	36.4 (16)	0.16
FSG (≥ 100 mg/dL)	27.3 (12)	63.6 (28)	0.001

Data are means ± SD. Significant values for p < 0.05. Chi-square test. MSyn: metabolic syndrome; WC: waist circumference; SBP: systolic blood pressure; DBP: diastolic blood pressure; TG: triglycerides; HDL-c: high-density lipoprotein; FSG: fasting serum glucose.

Being OMN increased the chance of having MSyn (OR: 5.79, 95%CI 2.13-15.76) and having altered different MSyn components: WC (OR: 6.80, 95%CI 2.62-17.70), SBP (OR: 2.83, 95% CI 1.13-7.12), DBP (OR: 4.38, 95%CI 1.53-12.53), TG (OR: 2.5, 95%CI 1.01-6.18), and FSG (OR: 4.67, 95%CI 1.89-11.52). Despite the higher risk of OMN developing CHD according to FSG, this difference was not shown in the logistic regression model (OR: 3.04, 95%CI 0.75-12.32).

The OMN diet was associated with a prevalence of MSyn (OR: 6.28, 95%CI 2.11-18.71) and alterations in most MSyn components [WC (OR: 7.54, 95%CI 2.55-22.29), SBP (OR: 3.06, 95%CI 1.06-8.82), DBP (OR: 4.08, 95%CI 1.27-13.07), and FSG (OR: 5.38, 95%CI 1.95-14.88)] in the multiple regression, independently of caloric intake, age, level of physical activity, and alcohol consumption (Table 4).

**Table 4:** Multivariate regression models of the association between type of diet and metabolic syndrome and its components

	OR	95%CI	p value	p value of the model
**MSyn**				
Vegetarian	1			
Omnivorous	6.28	2.11-18.71	0.001	0.006
Caloric intake	1.00	0.99-1.00	0.783	
Age	1.01	0.96-1.07	0.674	
Physically active	0.56	0.18-1.71	0.307	
Alcohol consumption	1.74	0.64-4.69	0.275	
**WC (≥ 90 cm)**				
Vegetarian	1			
Omnivorous	7.54	2.55-22.29	<0.001	<0.001
Caloric intake	0.99	0.99-1.00	0.700	
Age	1.01	0.96-1.08	0.636	
Physically active	0.66	0.21-2.04	0.470	
Alcohol consumption	3.04	1.11-8.25	0.029	
**SBP (≥ 130 mm Hg)**				
Vegetarian	1			
Omnivorous	3.06	1.06-8.82	0.039	0.006
Caloric intake	1.00	0.99-1.00	0.843	
Age	1.10	1.03-1.17	0.004	
Physically active	0.84	0.27-2.55	0.751	
Alcohol consumption	0.78	0.29-2.12	0.628	
**DBP (≥ 85 mm Hg)**				
Vegetarian	1			
Omnivorous	4.08	1.27-13.07	0.018	0.007
Caloric intake	1.00	1.27-13.07	0.018	
Age	1.09	1.02-1.16	0.012	
Physically active	0.99	0.31-3.17	0.986	
Alcohol consumption	1.32	0.45-3.86	0.617	
**TG (≥ 150 mg/dL)**				
Vegetarian	1			
Omnivorous	3.46	1.25-9.64	0.017	0.079
Caloric intake	0.99	0.99-1.00	0.293	
Age	0.99	0.93-1.04	0.611	
Physically active	0.35	0.11-1.07	0.066	
Alcohol consumption	1.68	0.66-4.30	0.280	
**FSG (≥ 100 mg/dL)**				
Vegetarian	1			
Omnivorous	5.38	1.95-14.88	0.001	0.005
Caloric intake	1.00	0.99-1.00	0.974	
Age	1.06	0.99-1.12	0.084	
Physically active	0.79	0.27-2.29	0.666	
Alcohol consumption	0.67	0.26-1.74	0.407	

OR: odds ratio; 95%CI: 95% confidence interval. Multiple logistic regression adjusted for caloric intake, age, physical activity, and alcohol consumption. MSyn: metabolic syndrome; WC: waist circumference; SBP: systolic blood pressure; DBP: diastolic blood pressure; TG: triglycerides; FSG: fasting serum glucose.

## Discussion

This study brought the scientific evidence that in apparently healthy men a VEG diet was associated with a lower percentage of individuals with MSyn compared to an OMN diet. This difference remained after the adjustment of other lifestyle characteristics, such as smoking, alcohol intake, and physical activity. In addition, FRS and other cardiovascular risk factors (CRF) were also lower in VEG subjects.

Our study is considered a pioneer for being the first to prove the association between the VEG diet and the development of MSyn in a population of Brazilian men, although an association has been reported between red meat consumption and an increase in the risk of developing MSyn after adjusting for confounders, in a cohort of Japanese-Brazilians.^28^

In the present study, VEG had significantly lower values for BMI, WC, SBP, DBP, TC, LDL-c, Apo b, TG, TC/HDL-c ratio, FSG, and HbA1c, which is in accordance with other studies around the world.

The Lima Study, conducted in Peru, with 45 OMN, 105 VEG, and 34 semi-vegetarians, has reported lower TC and LDL-c values in VEG compared to OMN.^1^ In a cross-sectional analysis of 773 subjects from the Adventist Health Study 2, in the United States, a VEG dietary pattern was associated with a more favorable profile for BMI, WC, SBP, DBP, TG, and FSG.^8^^,^^29^ Studies in the Brazilian population have found similar results to these of the present study. A study with OMN, VEG, and semi-vegetarians from the Adventist Church of São Paulo has found lower values for SBP, DBP, TC, and LDL-c in the VEG group.^5^

In another study comparing 56 VEG and 40 OMN in São Paulo, the VEG group had lower BMI and WC, but the levels of TG, TC, and LDL-c were equal between the groups, and VEG had higher HDL-c,^30^ in contrast to our study, in which HDL was similar between groups.

In addition, a few studies in the literature have evaluated the association of the VEG diet with FRS,^5^^,^^6^ which is an algorithm for assessing risk for CHD in the short term.^7^ In a study conducted with 67 VEG and 134 OMN, the MONICA Project, in the state of Espírito Santo, Brazil, blood pressure, FSG, TC, LDL-c, TG, and FRS were lower among VEG.^6^

In a sample of 391 female VEG and 315 OMN from Taiwan, the VEG status was associated with lower BMI, smaller WC, lower TC, LDL-c, HDL-c, TC/HDL-c and LDL-c/HDL-c ratios.^9^

Regarding the differences in CRF between VEG and OMN, one seems more consistent in the literature, the difference in blood pressure. In an elderly Taiwanese population, SBP was independently associated with VEG status,^11^ and a recent meta-analysis confirms that a VEG diet is associated with lower blood pressure.^4^

Despite scientific plausibility that can explain the impact of the higher fat content of the OMN diet on lipid metabolism, a large number of studies has found that glucose profile is improved by adopting a VEG diet. In a study conducted with a sample of 425 adults from the Isfahan Diabetes Prevention Study, a population-based prospective cohort in Iran, the VEG dietary pattern was inversely associated with the risk of abnormal FSG levels.^3^ In Taiwan, OMN had a greater risk of developing high FSG (HR: 1.16, 95%CI 1.02-1.32).^12^ In our study, similar differences were observed in indices evaluated in glucose metabolism.

We found no difference between the levels of HDL-c, but other studies have found statistical differences in this lipoprotein. Gadgil et al.^13^ have observed a higher amount of HDL-c in VEG Asian Indians living in the San Francisco Bay area. In São Paulo, VEG individuals have higher HDL-c too.^28^ In Taiwan, OMN individuals had a smaller risk of having lower HDL-c.^12^

A significantly greater number of VEG individuals was classified as physically active compared to OMN individuals. Data from the Elderly Nutrition and Health Survey in Taiwan (1999-2000) have shown that regular exercise was independently associated with VEG status.^11^ Pimentel has observed a higher tendency to be physically active among VEG individuals living in São Paulo, compared to OMN individuals.^30^ In this study, VEG individuals were more physically active; however, the observed differences in CRF were due to neither physical activity nor caloric intake.

In our study, the OMN diet was associated with the prevalence of MSyn and alterations in most of the MSyn components (WC, SBP, DBP, and FSG), independently of caloric intake, age, and level of physical activity. Some studies with different populations have shown this association, as discussed below.

A study aiming to verify the association of MSyn risk factors with selected markers of oxidative status (advanced glycation end products, advanced oxidation protein products) and microinflammation (C-reactive protein and leukocytes) in healthy OMN and VEG individuals has found that OMN individuals consumed significantly more protein, total fat, saturated as well as unsaturated fatty acids, and dietary cholesterol, and less dietary fiber; in addition, the VEG diet seems to exert beneficial effects on MSyn and risk factors associated with microinflammation.^10^

Rizzo et al.^8^ have observed in subjects from the Adventist Health Study 2 that a VEG dietary pattern was associated with a lower risk of MSyn, and this relationship persists after adjusting for lifestyle and demographic factors. For the female VEG individuals from a Buddhist hospital in Taiwan, the risks for MSyn were lower for ovo-lacto-vegetarians of 1-11 years and > 11 years, respectively, 45% and 42%, compared to OMN individuals after adjusting for other covariates.^9^

It should be noted that although the sample from the CARVOS study includes only men who are self-assessed as "healthy", many individuals were found to have MSyn among those who consumed an OMN diet. MSyn is defined by a constellation of interconnected physiological, biochemical, clinical, and metabolic factors that directly increase the risk of atherosclerotic cardiovascular disease, type 2 diabetes mellitus, and all-cause mortality. Lifestyle is one of the major predisposing factors of MSyn.^31^

In our study, the multivariate regression models show that the VEG diet was independently the best indicator of MSyn, and was associated with its components WC, SBP, DBP, and FSG, suggesting that the prevalence of MSyn could be due to the influences on its components. We hypothesize that the mechanism responsible for these differences exists in the composition of one’s diet. VEG subjects consume smaller amounts of total fat, saturated fat, and cholesterol, and larger amounts of unsaturated fat and fiber than OMN subjects do.^10^^,^^14^ The absence of red and processed meat intake could have an additional role.^14^

In our study, VEG and OMN individuals did not have a significant difference in caloric intake. VEG consumed significantly more carbohydrates, dietary fiber, and polyunsaturated fat. Moreover, OMN significantly ingested larger amounts of protein, total fat, saturated fat, and monounsaturated fat.

In addition, dietary patterns like VEG and the Mediterranean diet have a beneficial synergistic combination of antioxidants, fiber, potassium, magnesium, and phytochemicals,^31^ which may be responsible for the health benefits demonstrated in several scientific studies.

There are some limitations to this study. It had a relatively small number of subjects, and the cross-sectional design did not allow us to draw conclusions in terms of causal relationships. Future research must be conducted, especially prospective cohort studies in different populations to prove the impact of the vegetarian diet on the outcomes evaluated in this study.

The strength of our study is its highly homogenized sample, where all were nonsmokers, had no previous diagnosis of diabetes, dyslipidemia, cardiovascular or cerebrovascular diseases, hypertension or intake of antihypertensive medication, and had no difference in the frequency of alcohol beverage intake. The only difference between groups was our independent variable, diet, and physical activity, which was demonstrated but did not account for the differences found. In addition, we found in the same sampling a better profile of subclinical vascular disease evaluated by arterial stiffness, determined by carotid-femoral pulse wave velocity and carotid intima-media thickness, and distensibility in VEG compared to OMN subjects.^20^

Our study is important because its sample included only apparently healthy men, which is a large segment of society and, therefore, of great interest in terms of primary prevention of CVD. The study findings therefore will have a great positive impact in terms of public health economics and quality of life.

## Conclusion

This study provides evidence that, in apparently healthy men, a VEG diet is associated with lower levels of some CRF, as well as lower FRS and percentage of individuals with MSyn, suggesting a VEG diet can be considered a protective factor against the development of CVD.
